# Pathogenesis, Transmission, and Within-Host Evolution of Bovine-Origin Influenza D Virus in Pigs

**DOI:** 10.1155/2024/9009051

**Published:** 2024-05-14

**Authors:** Stéphane Gorin, Gautier Richard, Stéphane Quéguiner, Amélie Chastagner, Nicolas Barbier, Céline Deblanc, Séverine Hervé, Yannick Blanchard, Frédéric Paboeuf, Gaëlle Simon

**Affiliations:** ^1^Ploufragan-Plouzané-Niort Laboratory, Swine Virology Immunology Unit, French Agency for Food, Environmental and Occupational Health and Safety, Ploufragan, France; ^2^Ploufragan-Plouzané-Niort Laboratory, Viral Genetics and Biosecurity Unit, French Agency for Food, Environmental and Occupational Health and Safety, Ploufragan, France; ^3^Ploufragan-Plouzané-Niort Laboratory, SPF Pig Production and Experimentation, French Agency for Food, Environmental and Occupational Health and Safety, Ploufragan, France

## Abstract

Whereas bovine has been demonstrated as the main reservoir of influenza D virus (IDV), this virus was first isolated in a pig and is regularly detected in some swine populations. However, the role of swine in IDV ecology, as well as the outcomes of IDV infection in pigs, is still unclear. This study aimed to provide additional information on pathogenesis, transmission, and adaptation of a bovine-origin IDV in swine. An infection and transmission study, using an IDV strain isolated following a first passage on pig of a bovine IDV, was conducted on specific pathogen-free pigs, including inoculated and direct contact pigs. Two routes of inoculation were tested, i.e., nasal and tracheal. None of the inoculated or their contact pigs showed clinical signs, but all of them shed the virus in nasal secretions and seroconverted. Virus shedding started earlier in pigs inoculated intranasally as well as in their contact pigs, compared to pigs inoculated intratracheally and associated contacts, suggesting that the viral replication occurred preferentially in the upper respiratory tract. Sequencing data brought to light a mutation on hemagglutinin-esterase-fusion protein (L118F) in the bovine IDV-derived isolate obtained after the first passage on pig. This mutation was fixed in all viral strains obtained in this study, either from inoculated or contact pigs, and was maintained over the second and third passages on swine. The L118F mutation could be linked to the adaptation of the parental bovine IDV to the swine host and might have contributed to an efficient viral multiplication and subsequent pig-to-pig transmission.

## 1. Introduction

Influenza D virus (IDV) is the fourth genus of Influenzavirus, i.e., *Deltainfluenzavirus*, within the *Orthomyxoviridae* family. It was first identified from pigs in the USA in 2011 [1, 2]. Thanks to further serological and virological investigations, cattle is currently considered as a primary host and main reservoir for IDV [3–21]. IDV infections have also been evidenced in small ruminants [17, 20, 22, 23], horses [24], camelids [23, 25], as well as *suidae*, i.e., pigs [17, 26], feral swine [27], and wild boars [26]. The IDV genome was even detected in bioaerosol samples from poultry farms, but its presence in this environment could not be directly linked to an infection in poultry [28]. In addition, several studies showed seroconversion against IDV in humans with a prevalence of 1.3% in the USA and 26.1% in Italy reported among the general population [2, 29], when a very high prevalence (91%) was estimated among US cattle workers [30] and IDV genome was identified in nasal washes from dairy cattle workers [31], highlighting an occupational zoonotic transmission.

To gain knowledge on IDV pathogenesis in mammals and the associated potential zoonotic risk, experimentations have been conducted in cattle [32, 33], ferrets [2], pigs [2, 34], feral swine [27], guinea pigs [35], and mouse [36, 37]. A study was also carried out to assess interspecies transmission features between calves and pigs depending on the viral strain origin [38]. Experiments conducted in pigs demonstrate mild to moderate symptoms, but knowledge regarding intraspecies transmission and adaptation of IDV in swine remains scarce.

In this study, we conducted an experiment on specific pathogen-free (SPF) pigs (*Sus scrofa domesticus*) in order to learn more about pathogenesis, transmission, and adaptation of a bovine-origin IDV in swine. An IDV strain previously isolated from a pig experimentally infected with a bovine IDV [26] was inoculated through either nasal or tracheal routes in pigs. Naïve animals were placed in direct contact with the inoculated ones in order to evaluate intraspecies transmission and virus evolution during three passages on pig of the original bovine strain.

## 2. Materials and Methods

### 2.1. Virus Strains

D/bovine/Nebraska/9-5/2012 was previously propagated on swine testis (ST) cells to constitute the inoculum used in the first experimental inoculation of SPF pigs [26]. In this previous study, three 8-week-old SPF pigs were inoculated by tracheal route with 10^4.5^ TCID_50_ of D/bovine/Nebraska/9-5/2012. Thanks to this first passage on pig, IDV strain D/swine/France/150445/2015 (445/15) was isolated on ST cells from a nasal swab taken at day 13 (D13) postinoculation (pi) from the only pig that excreted virus particles. Swine IDV 445/15 was then further propagated on ST cells to produce the second inoculum used in this study.

### 2.2. Experimental Design and Sample Collection

**Figure 1:**
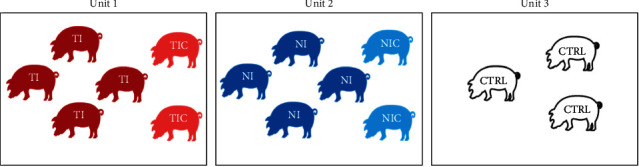
Experimental design. TI group (deep red) = pigs inoculated by the tracheal route; TIC group (light red) = pigs placed in direct contact to TI group; NI group (deep blue) = pigs inoculated by the nasal route; NIC group (light blue) = pigs placed in direct contact to NI group; CTRL = mock-inoculated pigs (Control group).

Fifteen 9-week-old SPF pigs were randomly assigned to five groups, depending on their route of infection, and housed in three separate air-filtrated BSL3 units (Figure 1). In Unit 1, four pigs were tracheally inoculated (TI group) and two pigs were placed in direct contact 6 hr postinoculation (hpi) (TIC group). In Unit 2, four pigs were nasally inoculated (NI group) and two pigs were placed in direct contact 6 hpi (TIC group). Each pig from TI and NI groups was inoculated with 10^6.5^ TCID_50_ in 5 ml of D/swine/France/150445/2015 strain. In Unit 3, three pigs were mock-inoculated with 5 ml of minimum essential medium (MEM) (CTRL group), two by the tracheal route and one by the nasal route.

Clinical signs were recorded daily from the day of inoculation (D0) to D28 and particular attention was paiyed to respiratory disorders or any influenza-like illness. Rectal temperatures were monitored daily from D0 to D28 in inoculated and contact groups, and at D0, 1, 3–4, 7−10, 13–21, 25, and 28 in the Control group. Body weight and food consumption were recorded twice a week until D11 (D0, D4, D7, and D11) and then weekly from D14 to D28 (D14, D21, and D28) in the three units. Blood samples were collected on dried tubes weekly from D0 to D28 in inoculated and contact groups in order to follow anti-IDV seroconversion, as well as at D0 and D28 in the Control group. To follow virus shedding, nasal swabs (MW 951 sent Virocult®, Corsham, UK) were taken daily from D0 to D18 in inoculated and contact animals and at D0 and D28 from controls. Air samples were collected at D2, D4, D7, D9, D11, D14, D16, and D18 in Units 1 and 2 using the Coriolis® Micro microbioal air sampler (Bertin Technologies, St-Quentin en Yvelines, France) (300 L/min, 10 min/room, in 15 ml of 0.005% Triton solution). Air sample eluates were obtained after a concentration step of 30 min at 3,900 g using Amicon® Ultra-15 30 K centrifugal filter devices (Merck Millipore Ltd., Ireland). All pigs were euthanized at D28 (TI, TIC, and CTRL) or D29 (NI and NIC) after anaesthesia (Zoletil®, Virbac, Carros, France, 10 mg/kg), followed by bleeding and then necropsied. Postmortem examination of the lungs was carried out, and tissue samples were collected and stored at −80°C until analyses.

### 2.3. Virus Detection by RT-qPCR

Viral RNA was extracted from cell culture supernatants, nasal swab supernatants, lung homogenates, sera, or air sample eluates using Nucleospin RNA^©^ or Nucleospin 8 RNA^©^ (Macherey Nagel, Hoerdt, France) according to manufacturer's instructions. Detection of the IDV PB1 gene was carried out in duplex with the porcine *β*-actin gene, as a housekeeping gene, as formerly described [26]. Forty PCR cycles were carried out, and all the samples with Cq values <40 were considered positive.

### 2.4. Virus Isolation and Titration

Virus isolation was attempted on ST cells for each inoculated or contact pig that was detected IDV positive by RT-qPCR. After 1h15 of incubation at 37° under stirring, nasal swab supernatants were eliminated, and cells were incubated with Dulbecco's modified Eagle's medium (DMEM) (Biowest, Nuaillé, France) supplemented with 1% penicillin–streptomycin (Dominique Dutscher, L0018-100) and 1 *µ*g/ml of tolysulfonyl phenylalanyl chloromethyl ketone (TPCK) treated trypsin (Whortington biochemical corporation, LS003740) at 37°C with 5% CO_2_ for 6 days. Virus growth was checked by the hemagglutination (HA) test using 0.5% chicken erythrocytes, complying with the WOAH standard protocols for swine influenza A viruses [39]. Titration was carried out by inoculating ST cells with 100 *µ*l of 10-fold serial dilutions of virus stock as described formerly [26]. The area under the curve (AUC) was calculated using virus excretion kinetics (TCID_50_/ml infectious titers) between D0 and D14 to evaluate global amounts of IDV infectious particles with GraphPad Prism (version 10.1.2). A zero baseline was taken into account, and the total area was calculated under the curve between the first X and last X different from zero for each pig. The AUC of the animals in the two contact groups was combined into a single contact group (TIC + NIC).

### 2.5. Hemagglutination Inhibition Assay

The hemagglutination inhibition (HI) assay for the detection of anti-IDV antibodies in swine serum was performed in accordance with the standard protocols applied for the detection of antibodies directed against swine influenza A virus, using D/swine/150445/2015 as an antigen and following the same procedure as formerly described [26]. Sera were considered positive when their titers were greater than or equal to 20. HI titers were log2-transformed in graphical representation.

### 2.6. IDV Genome Sequencing

IDV whole genome (seven segments) sequencing and/or sequencing of the hemagglutinin-esterase-fusion (HEF)-encoding gene were attempted on virus RNA extracted either from ST-propagated virus strains or from nasal swab supernatants. Next-generation sequencing (NGS) was performed on an Ion Proton instrument (ThermoFisher Scientific, Frederick, Maryland, USA). For whole genome sequencing, the cDNA libraries were prepared using the Ion Total RNA-Seq kit v2 (ThermoFisher Scientific). For HEF segment sequencing, amplicons of 2,042 bp size were first produced by RT-PCR using SuperScript III Platinum One-Step qRT-PCR (ThermoFisher Scientific) and HEF-specific primers [8]. The reverse transcription step was performed for 60 min at 42°C and, after an inactivation step of 2 min at 94°C, 5 cycles of 30 s at 94°C, 30 s at 45°C, and 3 min at 68°C were followed by 31 cycles of 30 s at 94°C, 1 min at 57°C, and 3 min at 68°C. Libraries were prepared using Ion Xpress Plus Fragment Library Kit (ThermoFischer Scientific). The raw reads were cleaned and first assembled by mapping on reference genomes using Burrows–Wheeler Aligner software (version 0.7.15-r1140) and, in addition, were *de novo* assembled using SPAdes (v3.10.0) and/or MIRA (v4.0.2) programs. The contigs produced by *de novo* methods were scaffolded and compared to the alignment on reference genomes to generate a single consensus sequence per viral segment using Vector NTI Advance 11.0 software (ThermoFisher Scientific). Analysis of single nucleotide polymorphism (SNP) in HEF sequence was performed with Varscan (v2.3.9). D/bovine/Nebraska/9-5/2012 strain (Accession numbers KM392468-KM392474) was used as a primary reference to identify the SNP.

### 2.7. Statistical Analyses

Average daily weight gain data were compared using nonparametric Kruskal–Wallis test with Holm's correction for pairwise comparisons to assess differences between groups. Virus titration data from TI and NI groups were compared in pairs with multiple Mann–Whitney tests, and the AUC of virus shedding kinetics obtained in the different groups was compared using ordinary one-way Anova and Tukey multiple comparisons test. Differences were considered significant when *p*-values (*p*) were less than 0.05. All statistical analyses were performed using R software (version 3.1.3) and GraphPad Prism (version 10.1.2).

### 2.8. Protein 3D Structure

The modelling of the D/swine/France/150445/2015 HEF protein 3D structure was performed and displayed using the SWISS-MODEL webserver [40] by uploading the D/swine/France/150445/2015 HEF sequence and finding a reference model (SMTL ID 5e64.2) based on amino acid identity. The obtained 3D model was then displayed in its hetero-3-3-mer oligo state with a colour-scheme based on the protein chains and as a zoomed view centred on the HEF open receptor-binding cavity with a colour-scheme based on amino-acid position. The notable secondary structures participating to the HEF receptor-binding site (RBS) were as described by Song et al. [41]. The numeration of the amino acids on the 3D model is shifted by 18 since the chosen reference lacks the first 18 amino acids.

## 3. Results

### 3.1. Clinical Signs and Virus Shedding

No clinical signs nor hyperthermia was observed during the course of the experiment, whatever the group (*Supplementary 1*). Moreover, the average daily weight gains were not statistically different between groups (*Supplementary 2*).

**Figure 2:**
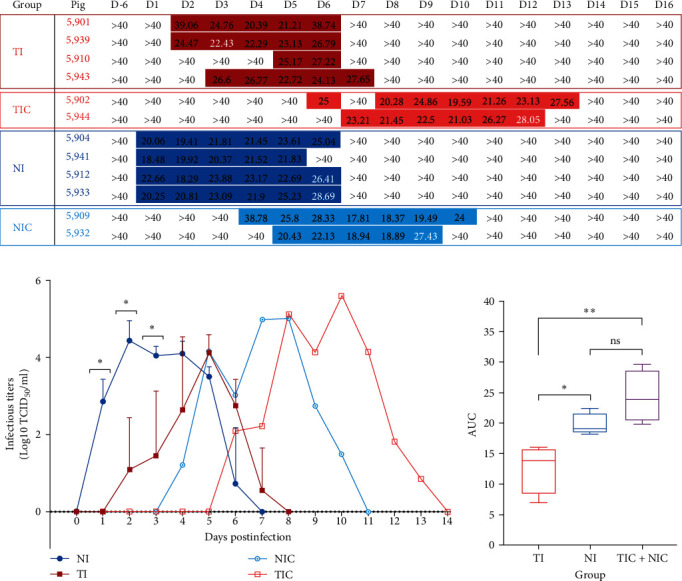
IDV shedding by inoculated and contact pigs. (a) Qualitative results of PB1-gene RT-qPCR on nasal swabs taken on pigs from D0 to D16. Parts with coloured background indicate the detection of IDV genome was possible. Ct values are shown in the coloured background sections. White background parts containing “>40” indicate that no viral genome was detected. Coloured background parts containing Ct values written in white font indicate that despite genome detection by RT-qPCR, viral titration was not possible. (b) IDV shedding in nasal secretion. Means (±standard deviation) of infectious titers (TCID_50_/ml log10-transformed) obtained in infected or contact groups over time after inoculation.  ^*∗*^Indicates that the NI group shed a significantly more important quantity of titrable virus particles than the TI group at D1, D2, and D3 (*p* value < 0.05). (c) Global amounts of IDV infectious particles shed from D0 to D14. Boxplot representations around the median for the TI group (deep red), NI group (deep blue), and TIC + NIC group (light purple).  ^*∗*^Indicates significantly different AUC with *p* value < 0.05, and  ^*∗∗*^indicates significantly different AUC with a *p* value < 0.005.

Virus shedding was measured by PB1-gene RT-qPCR in nasal swab supernatants (Figure 2(a)). In the TI group, all pigs shed the virus either from D2 (2/4) or D3 (1/4) or D5 (1/4), for 5 days for three of them, but only for 2 days for one of them. The two contact animals (TIC group) were infected as they shed the virus from D6 to D7, for 6–8 days. In the NI group, all the pigs shed the virus as soon as D1, for 5–6 days. The two contact animals (NIC group) were infected and shed the virus from D4 to D5, for 5–7 days. Control pigs remained negative throughout the entire experiment (data not shown). For a huge majority (60/65) of the samples in which IDV genome was detected, virus titration on ST cells was made possible. Four out of five samples from which virus titration was not possible were collected at the end of the apparent shedding period, corresponding to the last sampling day when IDV genome was detected by RT-qPCR. Comparisons of IDV excretion kinetics revealed that the amounts of infectious particles shed by pigs were significantly higher in the NI group than in the TI group at D1, D2, and D3 (*p* value < 0.05) (Figure 2(b), (*Supplementary 3*). Moreover, fewer variations between infectious titers among individuals were observed in the NI group as compared to the TI group. Besides, AUC comparisons showed that pigs inoculated by the nasal route shed more IDV infectious particles than those inoculated intratracheally (*p* value < 0.05) during the whole kinetics (Figure 2(c)). Since animals in TIC and NIC groups had a similar status regarding the route of inoculation, as they were both infected naturally, their data were combined into a single group for AUC calculation. AUC from this third group was significantly different from AUC of the TI group (*p* value < 0.005) but not of the NI group.

No viral genome was detected in bioaerosol samples, irrespective of the room and the air-sampling day. Note that viral genome was not detected in sera sampled at D7 and D14. Moreover, no lung injury was observed or viral genome detected in lung samples at necropsy at D28-D29, whatever the route of IDV infection, either tracheal/nasal inoculated or contact pig.

### 3.2. Seroconversion

**Figure 3:**
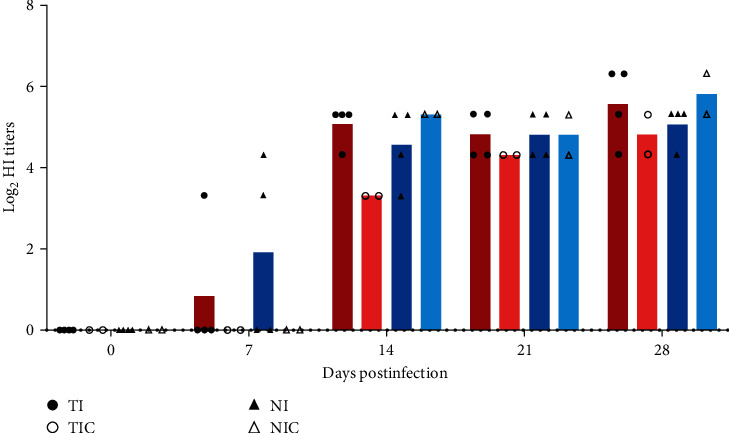
Anti-IDV antibody titers in sera from inoculated and contact pigs over time postinoculation. Mean hemagglutination (HI) titers (log2-transformed) are represented by coloured bars. Individual HI titres (log2-transformed) are represented by black dots of different shapes.

All inoculated and contact pigs seroconverted, as observed from HI tests performed on sera collected over time pi. At D7, sera from one of four pigs in the TI group and two of four pigs in the NI group displayed anti-IDV antibodies but none of the sera from contact pigs (Figure 3). At D14, anti-IDV antibodies were detected in all sera, whereas titers measured in those from the TIC group were markedly lower than others. At D21, all HI titers reached 20, i.e., the significant threshold of seroconversion (log2-transformed value = 4.32). At 28 dpi, antibody titers were maintained or continued to increase slightly, up to 80 (log2-transformed value = 6.32) at a maximum.

### 3.3. Within-Host Genetic Evolution

First, the parental D/bovine/Nebraska/9-5/2012 strain present in the inoculum used in our previous study [26], i.e., the cell-propagated bovine strain before any passage on pig (passage 0 on pig = P0), was submitted to WGS sequencing. When aligned to D/bovine/Nebraska/9-5/2012 sequence available under accession numbers KM392468-KM392474, five substitutions were observed in HEF ORF, and two of them led to amino acid mutations, i.e., A252V and G290R (*Supplementary 4*). These modifications could be linked to the sequencing or bioinformatics method used by Collin et al. [6] or have been fixed through further virus propagation on ST cells. The new D/bovine/Nebraska/9-5/2012 sequence has been deposited in GenBank under accession number PP417779-PP417785.

In any case, WGS sequencing of the P1 D/swine/France/150445/2015 strain, previously isolated after a first passage of the bovine IDV isolate on SPF pig (passage 1 on pig = P1) [26], showed it was 100% identical to the P0-inoculated strain D/bovine/Nebraska/9-5/2012 in all genes except the HEF-encoding one. Interestingly, a single nonsynonymous substitution (C to T) was observed in position 376 of the P1 strain when compared to the inoculated P0 strain at the nucleotide level, which translates to a L118F protein mutation (amino acid numbering from methionine). As D/swine/France/150445/2015 strain (P1) inoculum was obtained following two rounds of amplification on ST cells, we verified that it already exhibited the HEF-L118F substitution before this cell culture propagation. Thus, WGS was attempted on viral RNA extracted from cell culture supernatants (first passage on ST cells), as well as on RNA extracted from nasal swabs previously taken at D8, D10, and D13 on the pig from which it was isolated after initial inoculation of the D/bovine/Nebraska/9-5/2012 strain [26]. WGS confirmed that the D/swine/France/150445/2015 strain exhibited the substitution (in 95.31% of reads) after the first propagation step on ST cells. HEF gene sequencing implemented on these samples after specific DNA amplification showed that the C to T substitution in position 376 was present in the three samples (98.26%, 96.38%, and 98.96% of reads, respectively), demonstrating it was fixed as soon as the first passage of the bovine IDV on pig, previously to any propagation of the excreted virus on ST cells. HEF DNA sequencing performed on cell-propagated D/Bovine/Nebraska/9-5/2012 (unknown number of passages on ST cells) and D/Swine/France/150445/2015 (two passages on ST cells) also confirmed the L118F mutation in the swine IDV (96.64% of reads) compared to the bovine IDV (0% of reads). D/swine/France/150445/2015 strain sequence was deposited in GenBank under accession number PP417772-417778.

**Figure 4:**
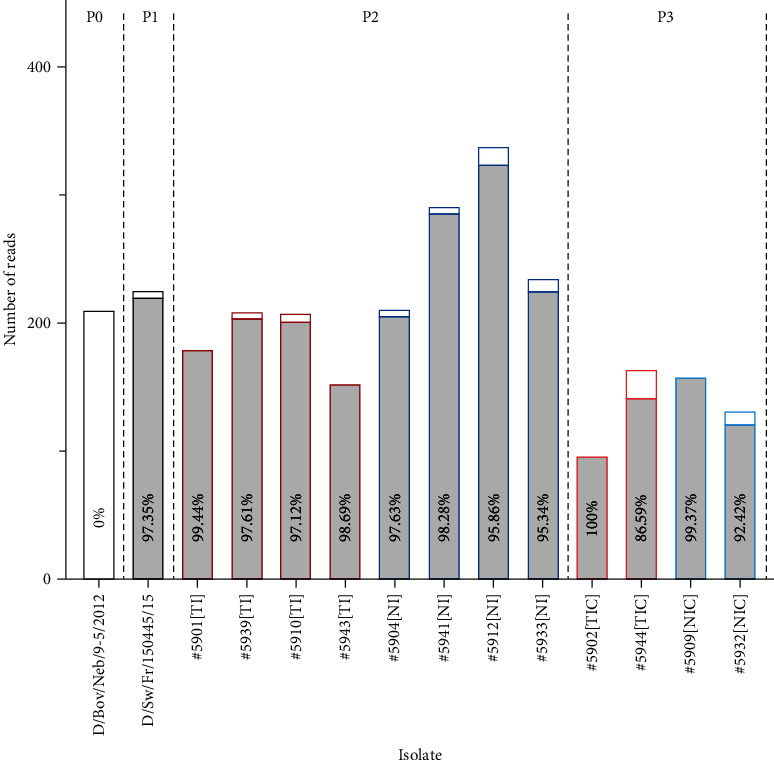
Numbers of reads (WGS) exhibiting the HEF-L118F substitution in deduced amino acid sequences of bovine IDV inoculum (P0) and swine IDV strains obtained after 1, 2, or 3 passages on pigs. Gray bars represent numbers of reads with L118F substitution, and white bars represent numbers of reads without this substitution. The percentages (%) are the proportions of HEF reads with L118F substitution. P0 = without passage on pig; P1 = first passage on pig; P2 = second passage on pig; P3 = third passage on pig.

Then, we submitted to WGS the twelve ST-propagated IDV isolates obtained in this study from nasal swab supernatants taken on excreting pigs. Eight isolates came from TI and NI-inoculated groups and counted for viruses obtained from a second passage of the parental bovine IDV on swine (passage 2 on pig = P2), whereas four others came from TIC and NIC contact groups, which means after a third passage of initial bovine IDV on pig (passage 3 on pig = P3). WGS of these eight P2 and four P3 strains revealed they were 100% identical to each other and 100% identical to the challenge strain D/swine/France/150445/2015 (P1 strain). After comparison to inoculated D/Bovine/Nebraska/9-5/2012 sequence (P0), we observed that the HEF-L118F mutation was present in more than 97% of reads from the P1 strain, in 97.50% (+/−1.38%) of reads from P2 strains, and in 94.60% (+/−6.35%) of reads from P3 strains, whereas it was absent in 100% of the reads obtained for the inoculated D/Bovine/Nebraska/9-5/2012 (P0) (Figure 4). Thus, the HEF-L118F mutation observed from the very first passage of bovine IDV on pig has been maintained in the second and third passages.

**Figure 5:**
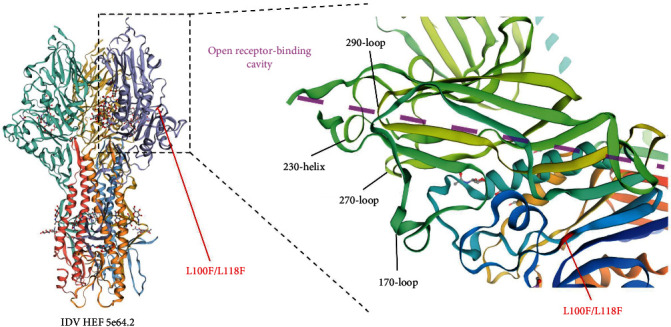
IDV HEF L118F mutation is close to the open receptor-binding cavity in the context of the HEF 3D structure. Left: entirety of the IDV HEF protein 3D model (reference 5e64.2) displayed as a trimer with the L118F mutation shown in red (L100F position in the context of the HEF 3D structure published by Song et al. [41]. Right: Zoom on the HEF open receptor-binding cavity (part of the HEF RBS) with the L118F (L100F) mutation displayed in red and the notable secondary structure of the HEF RBS according to Song et al. [41] shown in black.

The obtained HEF sequence of the P1 strain D/swine/France/150445/2015 was used to model the protein 3D structure (Figure 5). No major structural differences were found compared to reference IDV HEF sequences, devoid of the L118F mutation (data not shown). However, the L118F mutation carried by the P1 strain is close to the HEF open receptor-binding cavity.

## 4. Discussion

The first objective of this study was to compare the outcomes of IDV inoculation to pigs via either nasal or tracheal route. We observed that pigs can get infected in both cases. However, IDV excretion occurred earlier and was more homogenous and of higher intensity within the NI group compared to the TI group. Given that a nasal inoculation exposes the upper respiratory tract to the virus more than an intratracheal inoculation, these data may suggest that IDV could replicate preferentially in the upper respiratory tract. This assumption was sustained by excretion profiles of contact pigs, who became infected naturally. Indeed, pigs from the contact groups excreted significantly more virus than TI pigs, and as much as those in the NI group. This also suggested that IDV replicates better and is more easily excreted when it infects the upper respiratory tract. This is consistent with previous studies in pigs [2, 34, 38] and calves [32, 33] in which a preferential tropism of IDV for the upper respiratory tract was put forward. Nevertheless, we showed that infection through the tracheal route, which could better involve the lower respiratory tract as a replication site compared to the nasal route, is not a cul-de-sac, because all TI pigs excreted the virus, which was transmitted to and contaminated the contact (TIC) pigs. This is in line with other experimental studies that showed that IDV can also replicate in the middle and lower respiratory tracts of feral swine [27] and in both lower and upper respiratory tracts of guinea pigs [35].

We did not observe any difference between NIC and TIC groups regarding excretion profiles, except a delayed excretion for TIC animals that could be related to the later excretion of the TI pigs. Despite our contact groups were composed of only two pigs, which was a limitation to this experiment, it can be noted that IDV transmission was fully effective whether donors were inoculated by tracheal or nasal route. In previous studies, IDV transmission from pig-to-pig or from feral swine-to-feral swine or even from pig-to-calve was also reported to be effective but did not concern all individuals in the contact group [2, 27, 38].

Even though all animals became infected and excreted the virus for 6–7 days, none of them exhibited clinical signs, and growing performances were not affected. It cannot be ruled out that outcomes of infection would be different in pigs inoculated at other ages, as fever was previously observed in younger infected pigs [34]. No macroscopic lesion was observed at necropsy at D28/D29, and IDV genome was not detected in lung samples at that time, i.e., 4 weeks postinfection. Moreover, all animals seroconverted, indicating that the development of a humoral response certainly helped to neutralise the virus. In this study, we did not slaughter pigs at early time postinfection; thus, we were not able to compare virus multiplication in the lungs in the different groups. In another experiment conducted in pigs, IDV was not detected in lung samples taken at D7 post nasal inoculation, although the excretion peak in nasal secretion was demonstrated at D8 [2]. This also supports a preferential multiplication of IDV in the upper respiratory tract. However, in another experiment conducted on trapped feral swine, IDV was evidenced in the lungs from D3 to D10 although the inoculation was also carried out by the nasal route [27]. These differences could be linked to the animals, the virus strain, or the inoculating dose, but further analyses would be necessary to clearly identify the main site of IDV infection in swine.

Although outcomes of IDV infection in pigs were limited in this experimental study conducted on SPF pigs, IDV can cause mild symptoms in pigs [34], and coinfection studies with other respiratory pathogens should be interesting to evaluate its impact in the porcine respiratory complex. A study including *in vivo* and *in vitro* experiments reported a viral interference during a coinfection with IAV and IDV which was primarily linked to the proinflammatory response [42].

Even though we evidenced virus shedding in nasal secretion and virus transmission from donors to naive contact pigs, in accordance with Lee et al. [34], we failed in detecting IDV in air samples taken from D2 to D18 in both infected units. Yet, the same air sampling protocol has already proved its effectiveness in other experimental studies, allowing the detection of influenza A virus genome [43]. The low pig density, the large volume of air per individual, and the good renewal of air in the facilities, as well as the absence of coughing and sneezing postinfection, might explain that IDV detection in the air was not possible in this study. This did not exclude that aerosol transmission could have played a role in infection of contact pigs in this study, in addition to direct transmission through infected secretions. Bioaerosol transmission was demonstrated in a cattle experiment [33]; thus, further investigations including larger groups of inoculated pigs and indirect contact groups should be conducted to assess the potential role of aerosol transmission in the spread of IDV within, or even between, intensive pig herds.

We did not detect viral genome in sera samples at D7 and D14. Actually, IDV is not known to cause viremia in domestic pigs. Viremia was only highlighted on whole blood samples taken during an experimental study performed on trapped feral swine, in 4/12 inoculated pigs (at D3 and D5) and 1/12 of the contact animals (7 days post exposure) [27]. In another study, IDV was detected in sera samples from dairy cattle, hybrid white goat, and Asian buffalo [20]. However, most of these animals were known to have other health disorders or to be severe clinical cases sampled at the acute phase of the infection. Altogether, one may wonder if severe outcomes of IDV infection could promote viremia, which was not the case in our experiment.

In this study, pigs from NI and TI groups were inoculated with an IDV strain harvested from a pig previously infected with a bovine IDV strain [26]. In our formal experiment, the earliest IDV genome detection in nasal swabs occurred at D8 after tracheal inoculation of 10^4.5^ TCID_50_ of D/bovine/Nebraska/9-5/2012, in only one of the three inoculated pigs [27]. Maybe an intranasal inoculation of the bovine strain would lead to an earlier shedding as observed by others [38]. Here, the IDV genome was detected as early as D2 in 2–4 pigs inoculated intratracheally (TI group) with 10^6.5^ TCID_50_ of D/swine/France/150445/2015. While we cannot exclude that the higher inoculated dose may have accelerated the viral shedding in the second and third passages, the HEF-L118F mutation identified in the present study could have favoured virus entry and/or subsequent excretion as HEF is the only surface glycoprotein performing all entry and releasing functions, including receptor binding, receptor destroying, and fusion [2, 41]. This is reinforced by the proximity of the L118F mutation to the IDV HEF RBS and open receptor-binding cavity. This single-point mutation could be enough to allow a better adaptation of the virus to the swine host, as similar single-point mutations in HEF have already been shown to allow adaptation to other hosts, such as an HEF T284I mutation related to the adaptation of the JHB/1/66 influenza C strain to MDCK cells [44]. This mutation, which has never been described to date, neither in bovine nor swine IDV public sequences (data not shown), could be linked to an adaptation of the bovine strain to the swine species. As Kaplan et al. [38] suggested in a previous study, any genomic modification could contribute to phenotypic changes and help to cross the species barrier and promote interspecies transmission [38]. Further studies will be needed to verify this hypothesis, yet this substitution inevitably reinforces virus diversity.

## 5. Conclusion

In this study, we confirmed that, in domestic pigs, IDV most probably replicates more efficiently in the upper respiratory tract than in the lower one and showed IDV can be easily transmitted from pig-to-pig by direct contact. This supports the hypothesis that swine could play a significant role in IDV ecology, even in case of asymptomatic or mild infections. This is especially of importance in mixed pig-cattle herds, or pig herds in proximity to cattle, with pigs acting as potentially asymptomatic shedders able to spread IDV for instance beyond the species barriers and thus to act as a source of zoonosis, even if the public health relevance of IDV is still uncertain.

## Data Availability

The data used to support the findings of this study are included within the article.

## Supplementary Materials

Supplementary 1 Body temperature data taken daily from D0 to D18 and then at D21, D23, D25, and D28 for TI, TIC, NI, and NIC groups. For the Control group, temperatures were taken at D0, D1, D3, and D4, daily from D7 to D10, daily from D13 to D18, and then at D21, D23, and D25.

Supplementary 2 Daily weight gains calculated at different time intervals. No significant difference between groups compared in pairs at any time interval using the nonparametric Kruskal–Wallis test with Holm's correction for pairwise comparisons.

Supplementary 3 Infectious titers (TCID_50_/ml log10-transformed) from D0 to D14 postinoculation. Empty white boxes indicate that titration on ST cells has not been attempted. Grey-shaded cells indicate that titration was attempted but was not successful. The other cells contain the TCID_50_ infectious titer values calculated after titration on ST cells per millilitre of nasal swab supernatants.

Supplementary 4 Differences in the HEF sequence of D/bovine/Nebraska/9-5/2012 strain present in the P0 inoculum, inoculated to pigs in a first passage experiment [26], compared to the reference sequence published in databases.
