# Supplementary material for: Pathogenesis, Transmission, and Within-Host Evolution of Bovine-Origin Influenza D Virus in Pigs
Source: Transbound Emerg Dis. 2024 May 14;2024:9009051. doi: 10.1155/2024/9009051 (PMC12016950; doi:10.1155/2024/9009051)
Supplement: Supplementary 1 — Body temperature data taken daily from D0 to D18 and then at D21, D23, D25, and D28 for TI, TIC, NI, and NIC groups. For the Control group, temperatures were taken at D0, D1, D3, and D4, daily from D7 to D10, daily from D13 to D18, and then at D21, D23, and D25. [file 9009051.f1.pdf]

Group	Pig Number	D0	D1	D2	D3	D4	D5	D6	D7	D8	D9	D10	D11	D12	D13	D14	D15	D16	D17	D18	D21	D23	D25	D28
TI	5901	39.4	39.3	39.4	39.2	39	39	39.2	39.2	39.1	39.4	39.4	39.1	39.1	39	39.1	39.3	39.4	39.4	39.1	39.2	39.1	39.1	39.2
	5939	39.5	39.4	39	39.4	39.8	39.1	39.1	39.3	39.2	39.3	39.3	39	39.1	39.1	39.3	39	39.3	39.2	39.2	39	39.3	38.9	38.9
	5910	39.5	39.6	39.7	39.5	39.3	39	39.1	39.8	39.2	39.6	39.7	39.8	39.5	39.5	39.4	39.4	39.5	39.3	39.5	39.5	39.2	39.2	39.3
	5943	39.4	39.7	39.6	39.4	39.4	39.2	39.1	39.5	39.4	39.2	39.3	39.2	39.3	39.5	39.3	39.4	39.2	39.3	39.4	39.2	39.3	39.4	39.4
TIC	5902	39.1	39.7	39.8	40	39.6	39.2	39.3	40.1	39.2	39.6	39.9	39.5	39.3	39.5	39.3	39.6	39.3	39.5	39.5	39.4	39.5	39.4	39
	5944	39.5	39.1	39.2	39.2	39	39.1	39	39.2	39.6	39.3	39.3	39.4	39.3	39.1	39.2	39	39.2	39.2	39.1	39	39.2	39.4	39
NI	5904	39.3	39.5	39.2	39.6	39.1	39.4	39.1	39.3	39.4	39.4	39.4	39.4	39.3	39.2	39.4	39.2	39.3	39.1	39.3	39.4	39.4	39.4	39.2
	5941	39.4	39.5	39.4	39.5	39.2	39.2	39.1	39.3	39.4	39.4	39	39.2	39.1	39.4	39.2	39.1	39	39.2	39	39.1	39.4	39.3	39.1
	5912	39.6	39.3	39.6	39.7	39.5	39.1	39.4	39.6	39.3	39.2	39.1	39.3	39.3	39.3	39.2	39.4	39.3	39.5	39.3	39.3	39.2	39.1	39.1
	5933	39.8	39.8	39.5	39.7	39.6	39.2	39.2	39.7	39.2	39.5	39.5	39.5	39.3	39.2	39.3	39.2	39.5	39.3	39.5	39.5	39.3	39.4	39.4
NIC	5909	39.6	39.6	39.5	39.6	39.3	39.3	39.1	39.3	39.4	39.4	39.5	39.4	39.1	39.3	39.3	39.2	39.1	39.1	39.2	39.4	39.7	39.5	39.3
	5932	39.4	39.5	39.5	39.4	39.4	39.2	39.3	39.4	39.3	39.4	39.3	39.5	39.6	39.3	39.5	39.3	39.1	39.2	39.3	39.2	39.6	39.2	39.5
CTRL	5906	39.1	39.4		39.4	40.1			39.2	39.2	39.5	39.2			39.3	39.4	39.2	39.4	39.4	39.3	39		39.2	39.2
	5940	39.4	39.6		39.4	39.3			39.2	39.5	39.3	39.4			39.4	39.4	39.3	39.2	39.3	39.4	39.1		39.1	39
	5942	39.2	39.4		39.3	39.1			39.3	39.1	39.5	39.3			39.5	39.6	39.5	39.3	39.4	39.4	39.3		39.3	39.1
