# Supplementary figures and images for: Pathogenesis, Transmission, and Within-Host Evolution of Bovine-Origin Influenza D Virus in Pigs
Source: Transbound Emerg Dis. 2024 May 14;2024:9009051. doi: 10.1155/2024/9009051 (PMC12016950; doi:10.1155/2024/9009051)

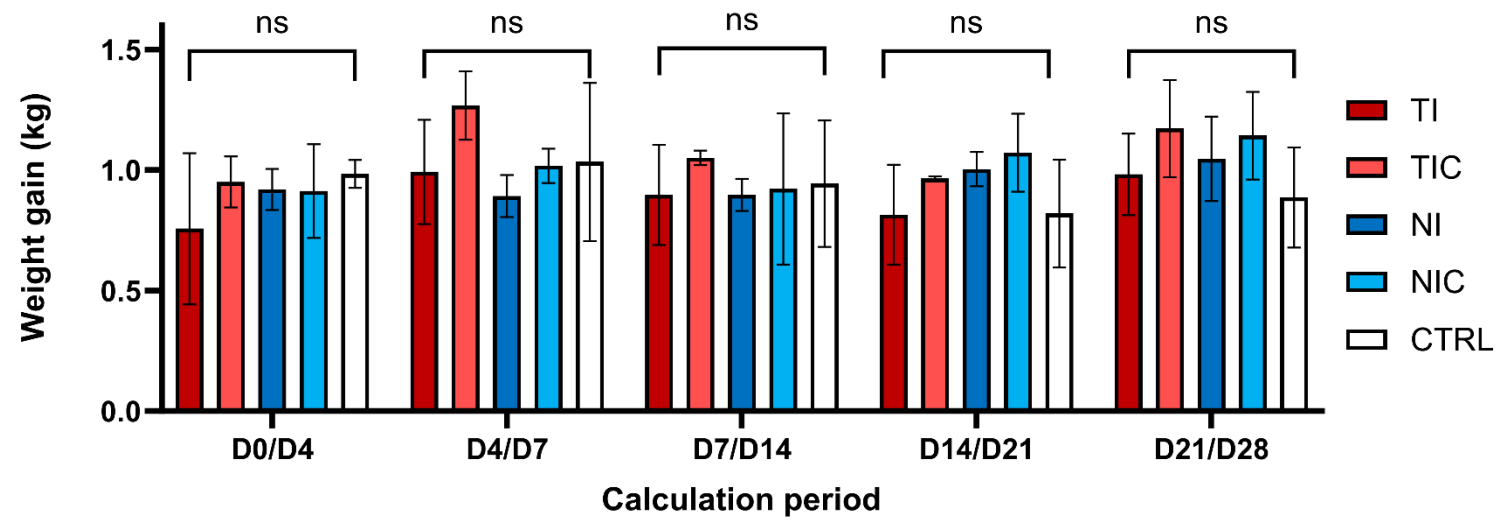

Supplement: Supplementary 2 — Daily weight gains calculated at different time intervals. No significant difference between groups compared in pairs at any time interval using the nonparametric Kruskal–Wallis test with Holm's correction for pairwise comparisons. [file 9009051.f2.pdf]
