# Supplementary material for: Pathogenesis, Transmission, and Within-Host Evolution of Bovine-Origin Influenza D Virus in Pigs
Source: Transbound Emerg Dis. 2024 May 14;2024:9009051. doi: 10.1155/2024/9009051 (PMC12016950; doi:10.1155/2024/9009051)
Supplement: Supplementary 3 — Infectious titers (TCID50/ml log10-transformed) from D0 to D14 postinoculation. Empty white boxes indicate that titration on ST cells has not been attempted. Grey-shaded cells indicate that titration was attempted but was not successful. The other cells contain the TCID50 infectious titer values calculated after titration on ST cells per millilitre of nasal swab supernatants. [file 9009051.f3.pdf]

Group	Pig Number	D0	D1	D2	D3	D4	D5	D6	D7	D8	D9	D10	D11	D12	D13	D14
TI	5901			5,13E+01	1,38E+03	1,48E+04	6,17E+04	1,55E+02								
	5939			5,13E+02		6,76E+03	1,00E+04	1,48E+02								
	5910						8,32E+03	1,00E+03								
	5943				4,90E+02	3,72E+02	6,76E+03	4,27E+03	1,74E+02							
TIC	5902							1,48E+04		3,63E+05	4,27E+03	4,90E+05	1,95E+05	4,27E+03	4,90E+01	
	5944								2,69E+04	4,90E+04	4,27E+04	3,16E+05	1,00E+03			
NI	5904		8,32E+02	2,09E+04	1,51E+04	3,16E+04	4,27E+03	7,94E+02								
	5941		1,48E+02	7,94E+04	1,95E+04	1,74E+04	2,75E+03									
	5912		5,13E+02	6,17E+04	8,32E+03	8,13E+03	5,62E+03									
	5933		3,63E+03	6,76E+03	6,76E+03	5,75E+03	1,48E+03									
NIC	5909					2,69E+02	3,16E+03	3,63E+01	6,17E+04	5,13E+04	3,16E+05	1,00E+03				
	5932						6,76E+04	3,16E+04	1,48E+05	2,04E+05						
