# Supplementary material for: Pathogenesis, Transmission, and Within-Host Evolution of Bovine-Origin Influenza D Virus in Pigs
Source: Transbound Emerg Dis. 2024 May 14;2024:9009051. doi: 10.1155/2024/9009051 (PMC12016950; doi:10.1155/2024/9009051)
Supplement: Supplementary 4 — Differences in the HEF sequence of D/bovine/Nebraska/9-5/2012 strain present in the P0 inoculum, inoculated to pigs in a first passage experiment [26], compared to the reference sequence published in databases. [file 9009051.f4.pdf]

**HEF-encoding gene (ORF) sequence**			**HEF protein sequence**			**Non-synonymous mutation** (yes/no)
Nucleotide number	KM392471 reference	P0 inoculum	Amino acid position	KM392471 reference	P0 inoculum	
747	C	T	252	A	V	yes
850	R	G	276	M/V	V	no
892	G	A	290	G	R	yes
947	R	G	308	R/K	R	no
1512	W	T	496	R/S	S	no
